# Effects of epigallocatechin-3-gallate combined with ascorbic acid and glycerol on the stability and uric acid-lowering activity of epigallocatechin-3-gallate

**DOI:** 10.1080/13880209.2021.1878235

**Published:** 2021-02-08

**Authors:** Qianjin Xie, Xiaqiang Cai, Xu Dong, Ying Wang, Minghui Sun, Lingling Tai, Yan Xu

**Affiliations:** a State Key Laboratory of Tea Plant Biology and Utilization, Anhui Agricultural University, Hefei, China; b International Joint Laboratory on Tea Chemistry and Health Effects of Ministry of Education, Hefei, China

**Keywords:** EGCG, Vc, stabilization, hyperuricaemia, serum UA

## Abstract

**Context:**

Epigallocatechin-3-gallate (EGCG) is unstable and easily oxidized, which limits its applications. Ascorbic acid (Vc) is a natural antioxidant.

**Objective:**

The effects of EGCG combined with Vc and glycerol on stability and uric acid-lowering activity of EGCG were examined.

**Materials and methods:**

EGCG (aqueous solution), EGCG + Vc (aqueous solution), EGCG (glycerol solution) and EGCG + Vc (glycerol solution) were prepared and incubated under different conditions *in vitro*. The recovery rate of EGCG was calculated by HPLC. Kunming mice were randomly divided into normal control group, model group, allopurinol (5 mg/kg), EGCG (10 mg/kg), EGCG + Vc (both 10 mg/kg), EGCG (10 mg/kg) + glycerol (60%), and EGCG (10 mg/kg) + Vc (10 mg/kg) + glycerol (60%) (*n* = 6). Allopurinol was injected intraperitoneally to mice, others were administered intragastrically to (2 cases) mice. All mice were continuously administrated for 7 days, once a day.

**Results:**

EGCG recovery rates of EGCG group and EGCG + Vc + glycerol group respectively reached to 32.34 ± 1.86% and 98.90 ± 0.64% when they were incubated for 4 h at 80 °C. EGCG recovery rates reached to 91.82 ± 5.13% (incubated for 6 h at pH 8) and 88.85 ± 2.63% (incubated for 4 h in simulated intestinal fluid) when EGCG incubated with Vc and glycerol. Compared with the model group, UA values of EGCG + Vc + glycerol group reduced by 43.49% while EGCG group reduced by 25.63%. The activities of xanthine oxidase (XOD, 31.41 U/gprot) and adenosine deaminase (ADA, 10.05 U/mgprot), and the mRNA expression levels of glucose transporter 9 (GLUT9, 1.03) and urate transporter 1 (URAT1, 0.44) in EGCG + Vc + glycerol group were notably lower than those of EGCG group (38.12 U/gprot, 13.16 U/mgprot, 1.54, and 0.55). The mRNA expression levels of ATP-binding cassette superfamily G member 2 (ABCG2, 1.39) and organic anion transport 1/2 (OAT1/2, 2.34, 2.53) in EGCG + Vc + glycerol group were notably higher than those of EGCG group (0.57, 1.13, and 1.16).

**Discussion and conclusions:**

Our findings suggest that when EGCG used in combination with Vc and glycerol could effectively increase its biology activities and can be generalized to the broader pharmacological studies. This sheds light on the development and application of EGCG in the fields of food and medicine.

## Introduction

EGCG is the most important biological active ingredient in green tea, accounting for more than 50% of the total amount of catechins (Nagle et al. 2006). Modern pharmacological research shows that EGCG has many physiological functions, such as antioxidation, anti-inflammation, anticancer, prevention of cardiovascular disease, antiviral, etc. (Masuda et al. 2002; Xu et al. 2008; Fu et al. 2017; Luo et al. 2017). But the clinical trials are not working well (Mereles and Hunstein 2011). The main reasons are that EGCG is unstable and easily oxidised and polymerised by light, oxygen, temperature, pH, and other factors *in vitro* (Zimeri and Tong 1999; Sang et al. 2005; Li et al. 2012). It is also easily degraded or polymerized *in vivo*. It has a short retention time and poor permeability in intestines and stomach. What's more, EGCG is more unstable and has very low bioavailability in blood environment (pH = 7.4) (Lambert et al. 2006; Krook and Hagerman 2012). Therefore, improving the stability of EGCG *in vivo* and *in vitro* is the pivotal factor to wide application of EGCG.

Vc is a natural antioxidant and free radical scavenger which can remove hydroxyl radicals (•OH), superoxide anion radicals (O_2_^−•^), etc. *in vivo* (Yang 2012). Glycerol is a broad-spectrum solubility polyol that is commonly used as a pharmaceutical excipient. Studies have found that some food or pharmaceutical ingredients when used in combination could improve their efficacy. For instance, EGCG combined with Vc can synergistically prevent the proliferation and invasion of 95-d cells in lung cancer (Yan et al. 2018). The aqueous glycerine solution combined with ginsenoside Rg1 can increase the absorption of ginsenoside Rg1 in the intestine and increase its absolute bioavailability in rats (Guo et al. 2017).

Hyperuricaemia is a metabolic disease. The serum UA levels of hyperuricaemia patients are higher than that of healthy persons, and its pathogenesis is related to purine metabolic disorder. The high level of uric acid in the body is not only the main reason for gout but also closely related to some diseases, such as hypertension (Kawano 2011; Mancia et al. 2015), coronary heart disease (Li M et al. 2016), kidney disease (Fathallah and Cramer 2014), metabolic syndrome (Borges et al. 2010), etc. Now, allopurinol (AP) (Schlesinger et al. 2009; Carro et al. 2010) and phenbromomarone are mainly used to cure hyperuricaemia in clinics, but they are often accompanied with many side effects and even lead to kidney failure and other hypersensitive reactions (Vazquez-Mellado 2001). Therefore, it is essential to develop natural and safe drugs for curing hyperuricaemia.

This paper explored the effect of EGCG combined with Vc and glycerol on the stability of EGCG at different temperatures, at different pH values and in simulated gastrointestinal fluids. Meanwhile the UA-lowering activity of EGCG combined with Vc and glycerol on hyperuricaemia mice was evaluated.

## Materials and methods

### Reagents and chemicals

EGCG was purchased from Jiangsu Dehe Biotechnology Co., Ltd. (the purity of EGCG was ≥95%). Pepsin, trypsin and Vc were purchased from Shanghai McLean Biochemistry Technology Co., Ltd. (China). Glycerol was purchased from Shanghai Titan Technology Co., Ltd. (China). Yeast extracts were purchased from Beijing Auberstar Biotechnology Co., Ltd. (China). Potassium oxonate (PO) was purchased from Nanjing Dulai Biotechnology Co., Ltd. (China). AP was obtained from Shanghai Sine Wanxiang Pharmaceutical Co., Ltd. (China). Biochemical Kits of UA, urea nitrogen (BUN), creatinine (Cr), xanthine oxidase (XOD) and adenosine deaminase (ADA) were purchased from Nanjing Jiancheng Bioengineering Co., Ltd. (China). Haematoxylin and Eosin (HE) Dyeing Kit was purchased from Boster Biological Technology Co., Ltd. (China). The HiScriptII 1st Strand cDNA Synthesis Kit and SYBR Green Master Mix Kit were obtained from Vazyme Biotech Co., Ltd. (China).

### Effect of Vc and glycerol on EGCG stability under different conditions

#### Determination of EGCG content

Chromatographic column was Luna 5 μ C18 (250 × 4.60 mm, 5 micron, Phenomenex, USA) with isocratic elution for 10 min by a mixture of 76% mobile phase A and 24% mobile phase B. The mobile phases were 0.17% acetic acid water (A) and acetonitrile (B). The UV detection wavelength was set at 278 nm and the column temperature was maintained at 30 °C. The injection volume was 10 μL at 1 mL/min flow rate. The recovery rate of EGCG was calculated base on the peak area of EGCG HPLC profiles. The formula was as follow:

EGCG recovery rate (%) =EGCG (after incubated)/EGCG (before incubated)×100

#### Effects of Vc and glycerol on EGCG stability under different temperatures conditions

EGCG (aqueous solution, 2 mg/mL), EGCG + Vc (aqueous solutions, both 2 mg/mL), EGCG (60% glycerol aqueous solutions, 2 mg/mL) and EGCG + Vc (60% glycerol aqueous solutions, both 2 mg/mL) were prepared. 1 mL of the above solutions was mixed with 9 ml buffer (pH = 7) solution, respectively.

They were incubated for 0, 1, 2, 3 or 4 h at 4, 37, 60 or 80 °C in the dark. The samples were analysed by HPLC.

#### Effects of Vc and glycerol on EGCG stability under different pH conditions

The initial concentration of phosphate buffer was 1 mol/L, then it was diluted to 0.2 mol/L and adjusted to pH 2, 4, 6 or 8 with HCl or NaOH. 1 mL of EGCG(aqueous solution, 2 mg/mL), EGCG + Vc (aqueous solutions, both 2 mg/mL), EGCG (60% glycerol aqueous solutions, 2 mg/mL) and EGCG + Vc (60% glycerol aqueous solutions, both 2 mg/mL) were mixed with 9 mL of different pH buffer solutions, respectively. They were incubated in the dark at 37 °C for 0, 1, 2, 4 or 6 h. The samples were analyzed by HPLC.

#### Effects of Vc and glycerol on EGCG stability under artificial simulated gastrointestinal fluid

The artificial simulated gastric fluid (ASGF) was prepared as follows: concentrated hydrochloric acid (9 mL) and pepsin (10 g) were dissolved in 800 mL water, the solution was diluted to 1000 mL with water, adjusting the pH to 1.3. The preparation method of artificial simulated blank gastric fluid (ASBGF) was the same as that of ASGF except for pepsin.

The artificial simulated intestinal fluid (ASIF) was prepared as follows: KH_2_PO_4_ (6.8 g) and trypsin (10 g) were dissolved in 500 mL water, the solution was diluted to 1000 mL with water, adjusting the pH to 7.6. The preparation method of the artificial simulated blank intestinal fluid (ASBIF) was the same as that of ASIF except for trypsin.

EGCG (1 mL) (aqueous solution, 2 mg/mL), EGCG + Vc (aqueous solutions, both 2 mg/mL), EGCG (60% glycerol aqueous solutions, 2 mg/mL) and EGCG + Vc (60% glycerol aqueous solutions, both 2 mg/mL) were mixed with 9 mL of ASGF, ASBGF, ASIF and ASBIF, respectively. They were incubated for 0, 1, 2, 3 or 4 h at 37 °C in the dark. The samples were analyzed by HPLC.

### Test animals

#### Establishment of hyperuricaemia mouse model

Kunming male mice [production licence No.: SCX (Beijing) 2016-0011], 25 ± 2 g of SPF, purchased from Beijing Weitong Lihua Experimental Animal Technology Co., Ltd. (China). Mice were fed for 1 week under the conditions of 12 h light/dark cycles, 23 ± 2 °C temperature and 50 ± 10% relative humidity. All the animals were humanly treated in accordance with the Guide for the Care and Use of Laboratory Animals (Ministry of Science and Technology of the People’s Republic of China) and the animal protocols were reviewed and approved by the ethics committee of Anhui Agricultural University.

Hyperuricaemia mice were established by intragastric administration of yeast extract (7.5 g/kg) and intraperitoneal injection of potassium oxonate (PO, 250 mg/kg). According to body mass, mice were housed in groups randomly (*n*= 6 each): normal control group (NC), model control group (MC), positive drug of AP group (aqueous solution, 5 mg/kg), EGCG group (aqueous solution, 10 mg/kg), EGCG + Vc group (aqueous solution, both 10 mg/kg), EGCG + glycerol group (60% glycerol aqueous solutions, 10 mg/kg) and EGCG + Vc + glycerol group (60% glycerol aqueous solutions, both 10 mg/kg). Before the experiment, mice were fasted for 12 h.

The NC group and the MC group were administrated intragastrically with saline, and the AP group were injected intraperitoneally with AP. The other groups were administered intragastrically with EGCG, EGCG + Vc, EGCG + glycerol and EGCG + Vc + glycerol, respectively. All mice were treated uninterruptedly for 7 days (Zhu et al. 2018; Tai et al. 2020). On the 7th day, 1 h before the administration, mice were orally administered with yeast extract and PO except for the NC group. After 1 h of the last administration, the chloral hydrate anaesthetic was injected intraperitoneally into mice. Under anaesthesia, the blood samples were collected from the orbital veins. Then mice were sacrificed by cervical dislocation, and the hepatic and renal tissues were taken out on the iced-bath and stored for future use. The kidney tissues were separated into two parts: one part was stored in RNA store reagent and the other part was stored in 10% formaldehyde solution.

#### Detection of biochemical indicators

The blood samples were placed at room temperature for 1 h and centrifuged at 4 °C, 5000 rpm for 10 min. The serum UA, BUN, and Cr values were analyzed according to the instructions of kits.

The liver tissues were homogenized and centrifuged at 3000 rpm for 10 min at 4 °C. The supernatant liquids were used for analysis of the activities of ADA and XOD. They were measured using kits.

#### Analysis of renal GLUT9, URAT1, ABCG2, and OAT1/2 mRNA expression levels and preparation of renal tissue sections

The mRNA expression levels of renal glucose transporter 9 (GLUT9), urate transporter 1 (URAT1), ATP-binding cassette superfamily G member 2 (ABCG2) and organic anion transport 1/2 (OAT 1/2) were analysed by qRT-PCR, according to the kits’ instructions. Fluorescence was quantified using mouse kidney tissue cDNA as template and GAPDH as internal reference gene. The relative quantification was calculated by 2^−ΔΔCt^ method.

Kidney tissues were taken out from 10% formaldehyde solution, washed thoroughly with PBS buffer (0.1 mol/L, pH 7.4), dehydrated in 70, 80, 90, or 100% ethanol, transition with xylene, embedded in paraffin and cut into 4 μm thick tissues sections. The sections were stained with haematoxylin and eosin (HE) and observed using optical microscope at magnification of 400×.

### Statistical analysis

The experimental data were calculated by Excel and expressed by average ± standard deviation ($\bar{\text{X}}$ ± SD). They were statistically analyzed using Graph Pad Prism 5 software to obtain histograms and carried on the difference analysis. Significant differences between groups were judged by one-way analysis of variance (ANOVA), followed by Student’s *t-*test. Differences were significant when *p* < 0.05.

## Results

### Effects of Vc and glycerol on the stability of EGCG at different temperatures

**Figure 1.**
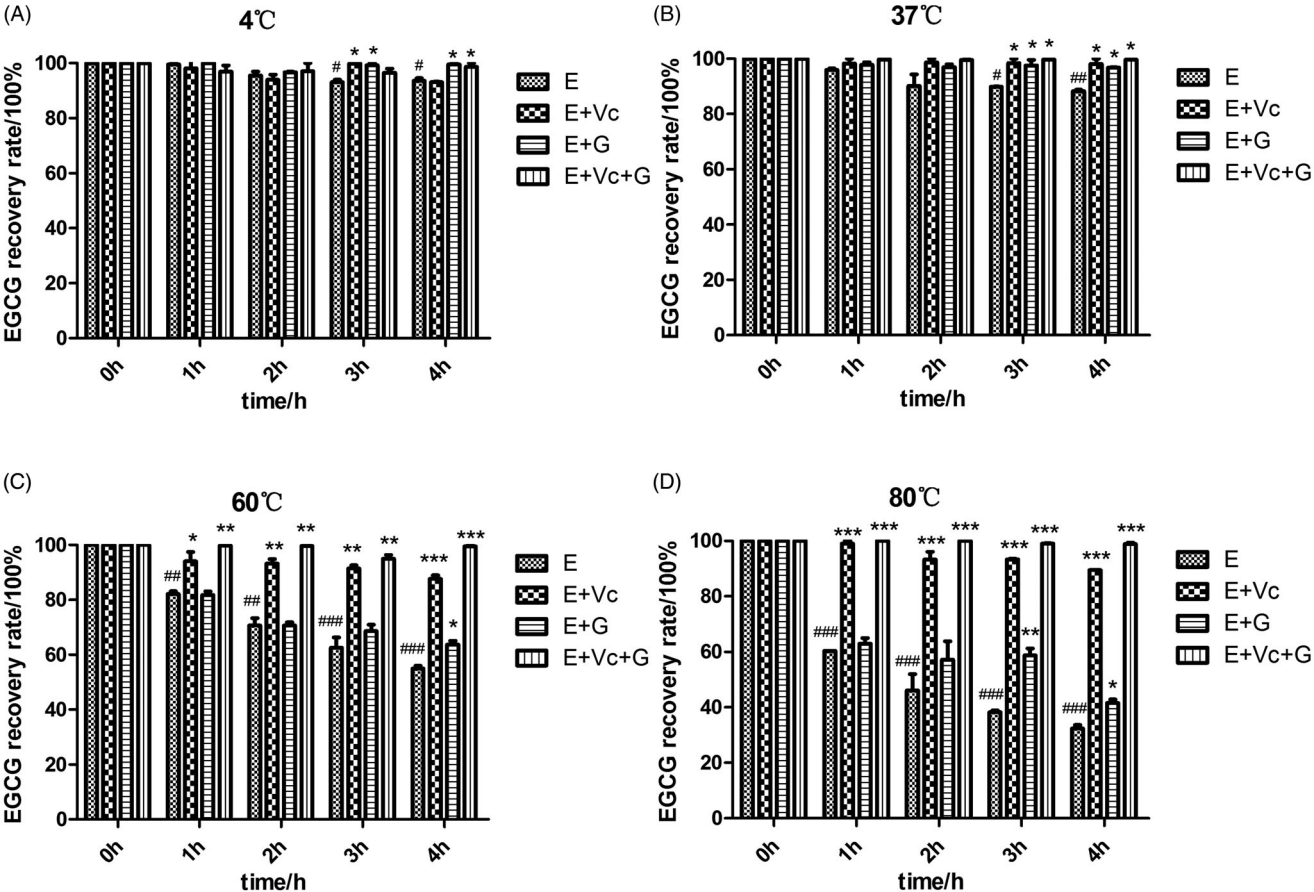
Effects of Vc and glycerol on the stability of EGCG incubated in the dark at different temperatures (*n* = 3). *p < 0.05, **p < 0.01, and ***p < 0.001 means compared with EGCG at the same time and temperature. #*p* < 0.05; ##*p* < 0.01; ###*p* < 0.001 represents compared with EGCG of 0 h at the same temperature.

The free EGCG incubated at 4 and 37 °C was relatively stable than incubated at 60 and 80 °C. The recovery rate of EGCG was 93.10 ± 1.23% and 88.26 ± 0.71% after incubated for 4 h at 4 and 37 °C, respectively (*p* < 0.05 or *p* < 0.01) (Figure 1(A,B)), while its recovery rate was 54.97 ± 1.44% and 32.34 ± 1.86% after incubated for 4 h at 60 and 80 °C, respectively (*p* < 0.001) (Figure 1(C,D)).

Compared with the EGCG group, Figure 1 also showed that EGCG + Vc and EGCG + Vc + glycerol groups could increase the EGCG recovery rate remarkably after they were incubated for 1, 2, 3, and 4 h at 60 and 80 °C (*p* < 0.05, *p* < 0.01 or *p* < 0.001). The EGCG + glycerol group showed significant protective effects on EGCG after they were incubated for 4 h at 4, 37, 60 and 80 °C (*p* < 0.05). The data showed that EGCG combined with Vc or/and glycerol could increase the recovery rate of EGCG obviously at different temperatures. The protection effect of EGCG combined with Vc and glycerol on EGCG was better than that of other groups. And the EGCG recovery rate of EGCG combined with Vc and glycerol could reach to 98.90 ± 0.64% after incubated for 4 h at 80 °C.

### Effects of Vc and glycerol on the stability of EGCG at different pH values

**Figure 2.**
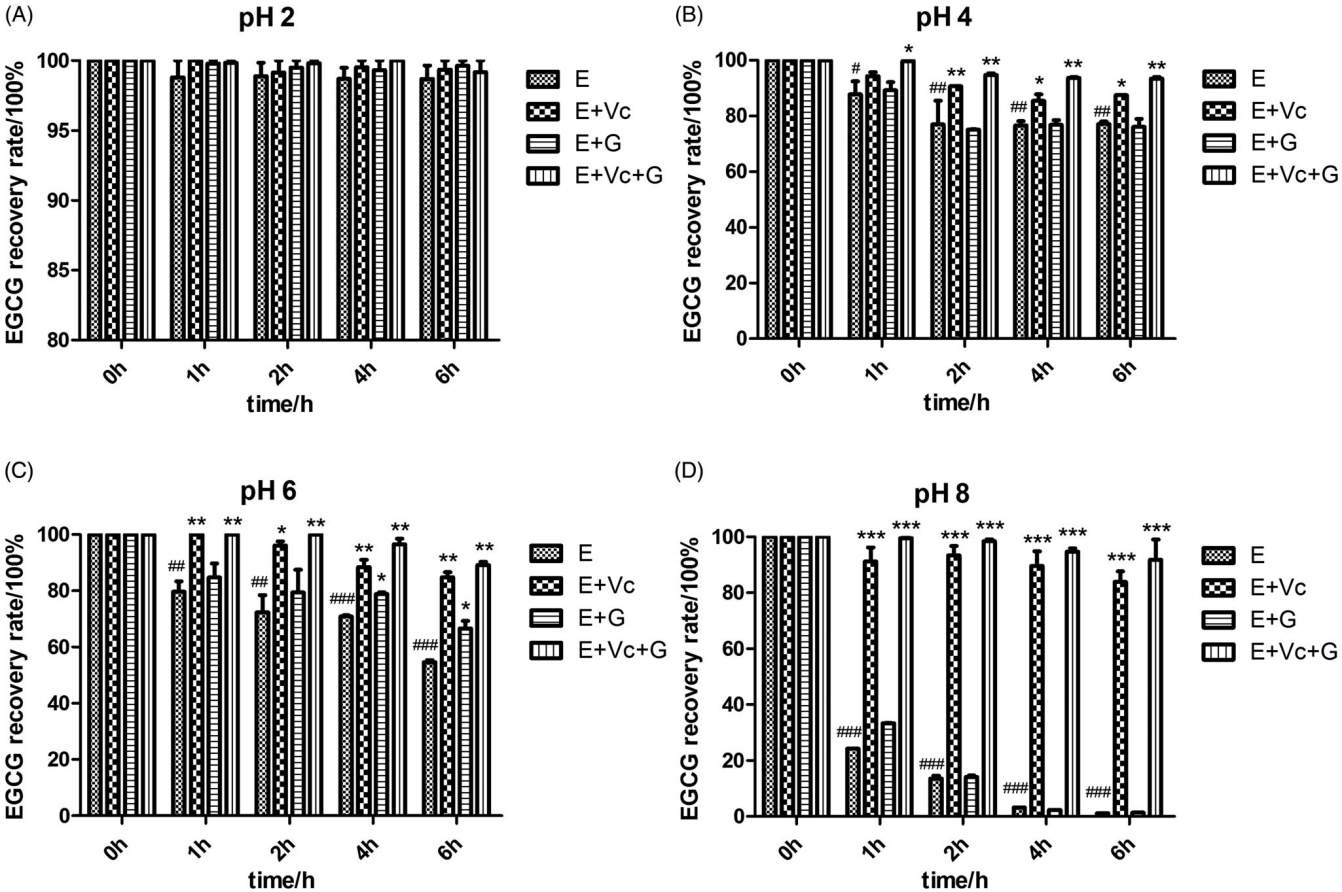
Effects of Vc and glycerol on the stability of EGCG incubated in the dark at 37 °C with different pH values (*n* = 3). *p < 0.05, **p < 0.01, and ***p < 0.001 means compared with EGCG at same time. #*p* < 0.05, ##*p* < 0.01, and ###*p* < 0.001 expresses compared with EGCG at 0 h.

Figure 2(A) revealed that the free EGCG was very stable at pH 2 and its recovery rate was 98.69 ± 1.36% after 6 h, but its recovery rate dropped down notably with pH values increasing. The recovery rate of EGCG was 77.12 ± 1.25%, 54.66 ± 1.06% and 1.11 ± 0.07% after they were incubated for 6 h at pH 4, 6, and 8 (Figure 2(B–D)).

Compared with EGCG group, Figure 2 also showed that EGCG + Vc and EGCG + Vc + glycerol groups could significantly increase the EGCG recovery rate after they were incubated for 2, 4, or 6 h at pH 4, 6 or 8 (*p* < 0.05, *p* < 0.01 or *p* < 0.001). EGCG + glycerol could obviously increase the EGCG recovery after incubated for 4 or 6 h at pH 6 (*p* < 0.05). The results showed that EGCG + Vc and EGCG + Vc + glycerol could improve the EGCG stability remarkably at different pH values. The recovery rate of EGCG combined with Vc and glycerol reached to 91.82 ± 5.13% after it was incubated for 6 h at pH 8. The protection effect of EGCG combined with Vc and glycerol on EGCG was superior to other groups.

### Effects of Vc and glycerol on the stability EGCG under artificial simulated gastrointestinal fluids

**Figure 3.**
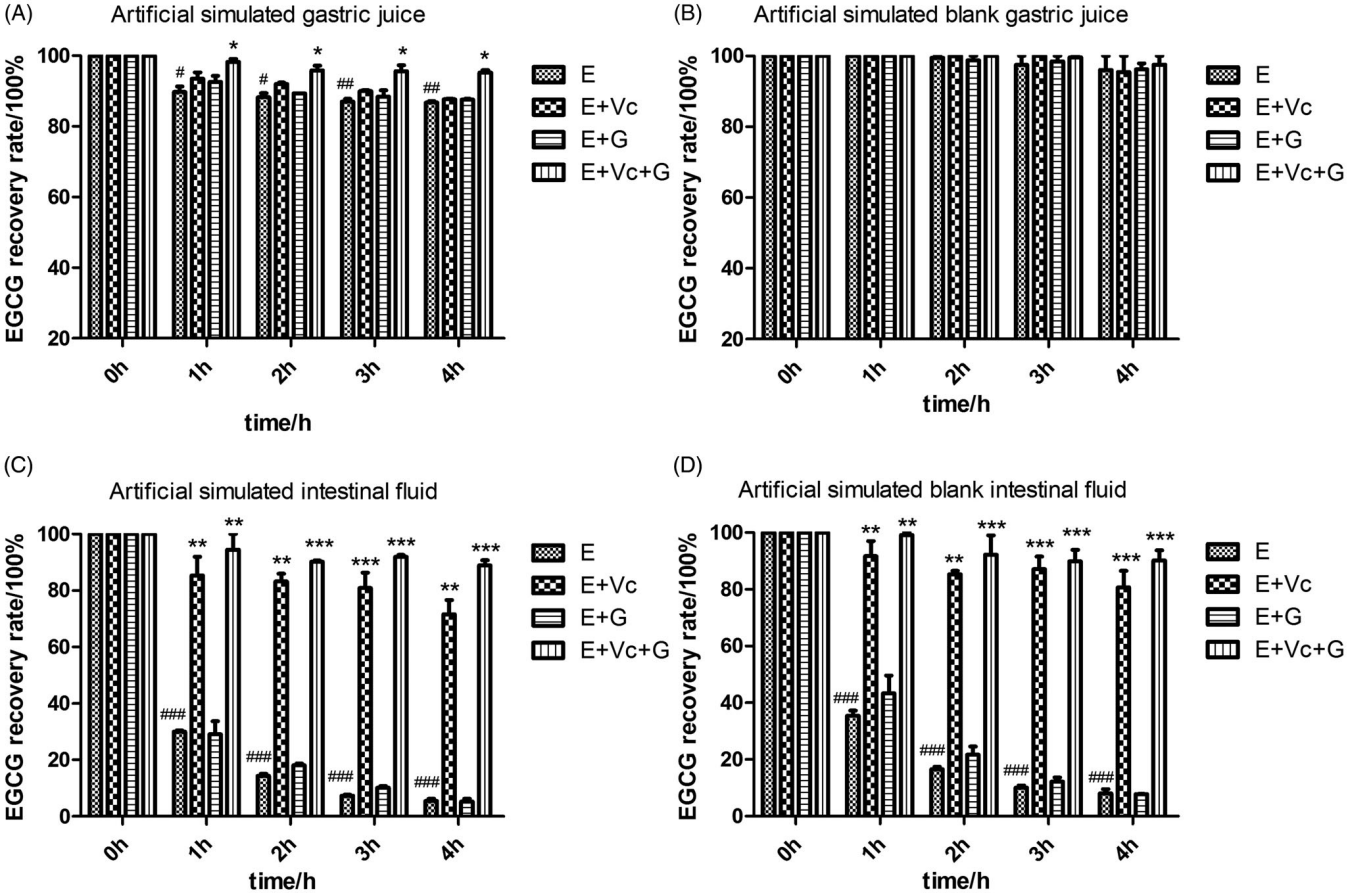
Effects of Vc and glycerol on the stability of EGCG in artificial simulated gastrointestinal fluids incubated at 37 °C in the dark (*n* = 3). *p < 0.05; **p < 0.01, and ***p < 0.001 means compared with EGCG at same time. #*p* < 0.05, ##*p* < 0.01, and ###*p* < 0.001 represents compared with EGCG at 0 h.

Figure 3(A,B) revealed that the free EGCG was stable in ASBGF but was unstable in ASGF. The EGCG recovery rate of EGCG group decreased to 86.74 ± 0.62% after incubated for 4 h in ASGF, while its recovery rate in EGCG + Vc + glycerol group was up to 95.23 ± 1.09%. There was significant difference between them (*p* < 0.05).

Figure 3(C,D) revealed the free EGCG was not stable in ASIF and ASBIF. The EGCG recovery rates of EGCG groups dropped to 14.38 ± 1.10% and 16.50 ± 1.37% in ASIF and ASBIF after incubated for 2 h, respectively. However, the EGCG recovery rates of EGCG + Vc and EGCG + Vc + glycerol groups were obviously higher than that of EGCG and EGCG + glycerol groups in ASIF and ASBIF (*p* < 0.01 or *p* < 0.001). The EGCG recovery rate of EGCG + Vc + glycerol group was up to 88.85 ± 2.63% and 90.19 ± 5.12% after incubated for 4 h in ASIF and ASBIF, respectively. The results indicated that the protective effect of EGCG combined with Vc and glycerol on EGCG were better than that of EGCG, EGCG + glycerol and EGCG + Vc groups in ASIF and ASBIF.

### Effect of EGCG combined with Vc and glycerol on serum UA in the hyperuricemic mice

**Figure 4.**
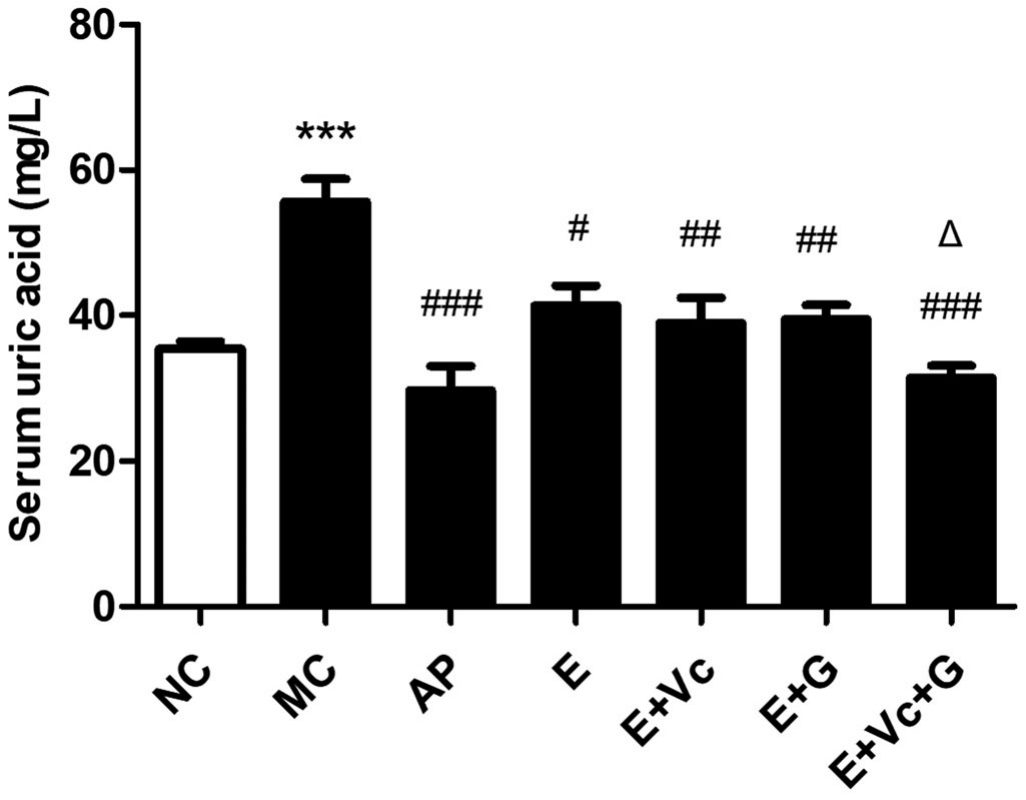
Effect of EGCG combined with Vc and glycerol on serum UA in the hyperuricemic mice. Values are means ± SE (*n* = 6). ***p < 0.001 when compared with the NC group; #*p* < 0.05, ##*p* < 0.01, ###*p* < 0.001 when compared with the MC group. Δ*p* < 0.05 when compared with the E group.

Figure 4 showed that the serum UA values of MC group were notably higher than that of NC group (*p* < 0.001). Compared with MC group, the serum UA values of AP, EGCG, E + Vc, E + G and E + Vc + G groups all decreased significantly (*p* < 0.05, *p* < 0.01 or *p* < 0.001). Meanwhile, there was significant difference between EGCG and E + Vc + G groups (*p* < 0.05), but there was no significant difference between EGCG, E + Vc and E + G groups. The results indicated that the UA-lowering activity of EGCG combined with Vc and glycerol was better than that of EGCG, E + Vc and E + G groups.

### Effect of EGCG combined with Vc and glycerol on serum BUN and Cr in the hyperuricemic mice

**Figure 5.**
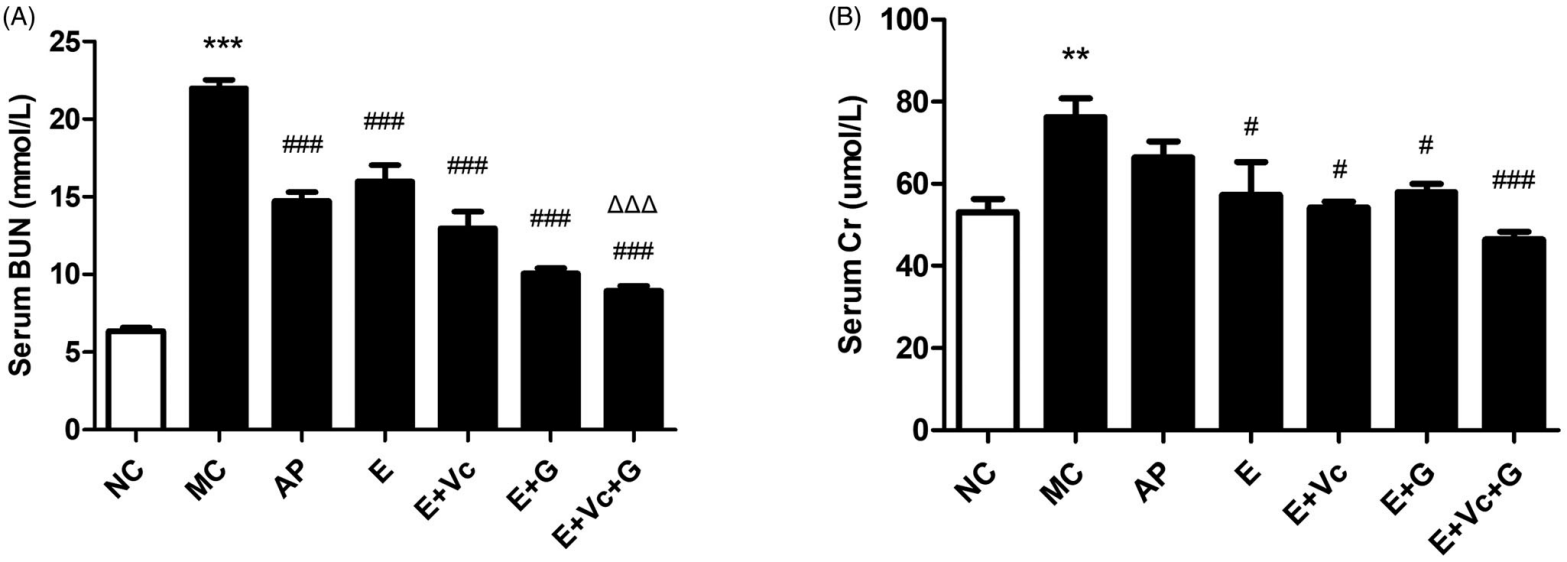
Effect of EGCG combined with Vc and glycerol on serum BUN (A) and Cr (B) levels in the hyperuricemic mice. Values are means ± SE (*n* = 6). **p < 0.01, ***p < 0.001 when compared with the NC group; #*p* < 0.05, ###*p* < 0.001 when compared with the MC group. ΔΔΔ*p* < 0.001 when compared with the E group.

Figure 5 revealed that the serum BUN and Cr levels were significantly difference between MC and NC groups (*p* < 0.01or *p* < 0.001). Compared with MC group, the serum BUN and Cr of EGCG, E + Vc, E + G and E + Vc + G groups were all reduced remarkably (*p* < 0.05 or *p* < 0.001). Furthermore, the serum BUN values of E + Vc + G group were significantly lower than that of EGCG group (Figure 5(A)) (*p* < 0.001). However, their serum Cr values had no significant difference (Figure 5(B)). The results displayed that the serum BUN and Cr of E + Vc + G group were lower than that of other groups.

### Effect of EGCG combined with Vc and glycerol on the activities of hepatic ADA and XOD in the hyperuricemic mice

**Figure 6.**
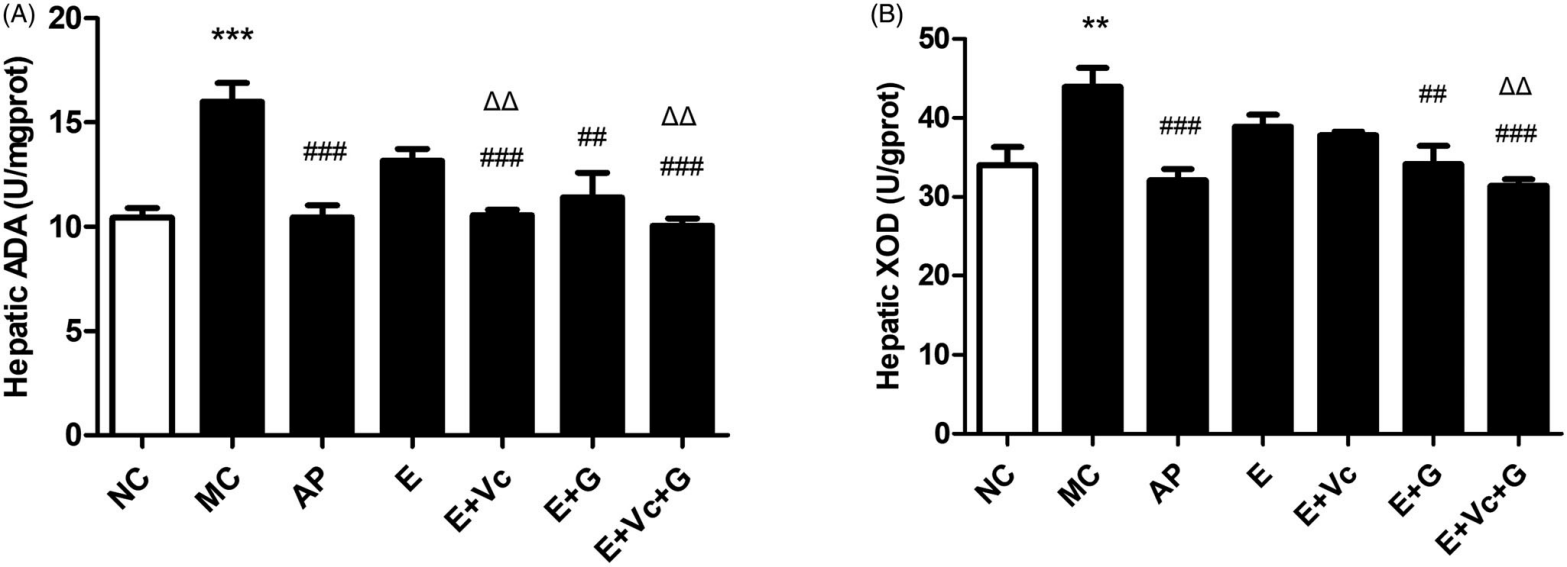
Effect of EGCG combined with Vc and glycerol on the activities of hepatic ADA (A) and XOD (B) in the hyperuricemic mice. Values are means ± SE (*n* = 6). **p < 0.01, ***p < 0.001 when compared with the NC group; ##*p* < 0.01, ###*p* < 0.001 when compared with the MC group. ΔΔ*p* < 0.01 when compared with the E group.

Figure 6(A) demonstrated that the ADA activities of AP, EGCG, E + Vc, E + G and E + Vc + G groups were suppressed remarkably compared with MC group (*p* < 0.01 or *p* < 0.001). The inhibition of E + Vc, E + Vc + G groups on ADA activity was obviously better than that of EGCG group (*p* < 0.01). As shown in Figure 6(B), the XOD activity of EGCG group was lower than that of MC group, but there was no significant difference. The XOD activities of E + Vc + G group were notably lower than that of EGCG group (*p* < 0.01). These indicated that EGCG combined with Vc and glycerol could significantly inhibit the activities of ADA and XOD in hyperuricemic mice compared with EGCG group.

### Effect of EGCG combined with Vc and glycerol on the mRNA expression levels of renal GLUT9, URAT1, ABCG2, and OAT1/2 in the hyperuricemic mice

**Figure 7.**
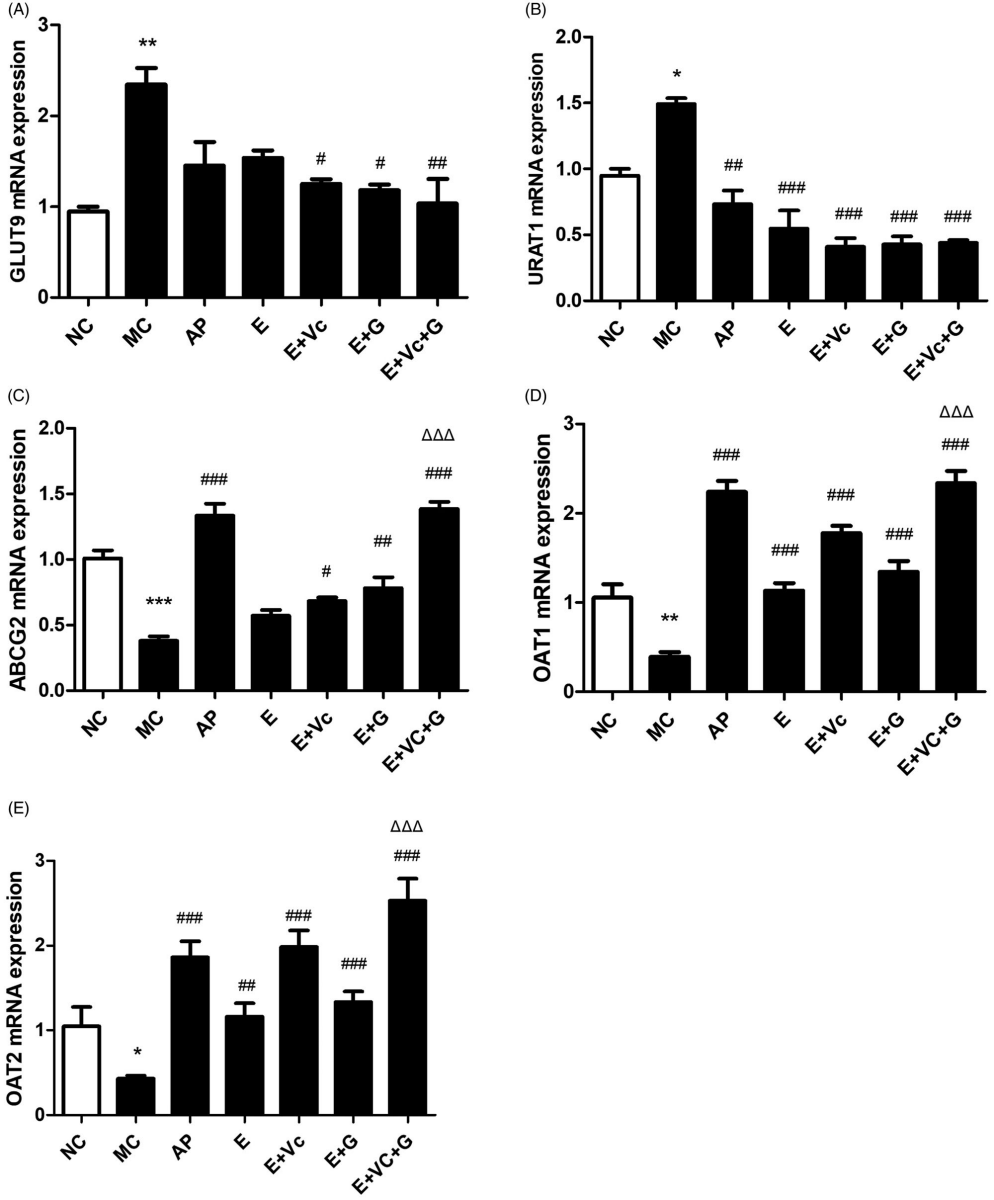
Effect of EGCG combined with Vc and glycerol on the mRNA expression of renal GLUT9, URAT1, ABCG2, and OAT1/2 in the hyperuricemic mice. Values are means ± SE (*n* = 6). *p < 0.05, **p < 0.01, ***p < 0.001 when compared with the NC group; #*p* < 0.05, ##*p* < 0.01, ###*p* < 0.001 when compared with the MC group. ΔΔΔ*p* < 0.001 when compared with the E group.

Figure 7(A) showed that the mRNA expression levels of renal GLUT9 in E + Vc, E + G and E + Vc + G groups were significantly down-regulated compared with MC group (*p* < 0.05 or *p* < 0.01). Figure 7(B) showed that the mRNA expression levels of renal URAT1 in drug-treated groups were all significantly down-regulated compared with MC group (*p* < 0.01 or *p* < 0.001). The mRNA expression levels of renal GLUT9 and URAT1 of E + Vc, E + G and E + Vc + G groups were lower than that of EGCG group, but there was no significant difference among them. The mRNA expression levels of renal ABCG2 and OAT1/2 in drug-treated groups were all obviously up-regulated compared with MC group except ABCG2 of the E group (shown in Figure 7(C–E)) (*p* < 0.05, *p* < 0.01 or *p* < 0.001). The mRNA expression levels of renal ABCG2, and OAT1/2 of E + Vc + G group were remarkably higher than that of EGCG group (*p* < 0.001).

These indicated that EGCG combined with Vc and glycerol could help EGCG down-regulate the mRNA expression levels of renal GLUT9 and URAT1, and up-regulate the mRNA expression levels of renal ABCG2, and OAT1/2.

### Morphological examination of the kidneys

**Figure 8.**
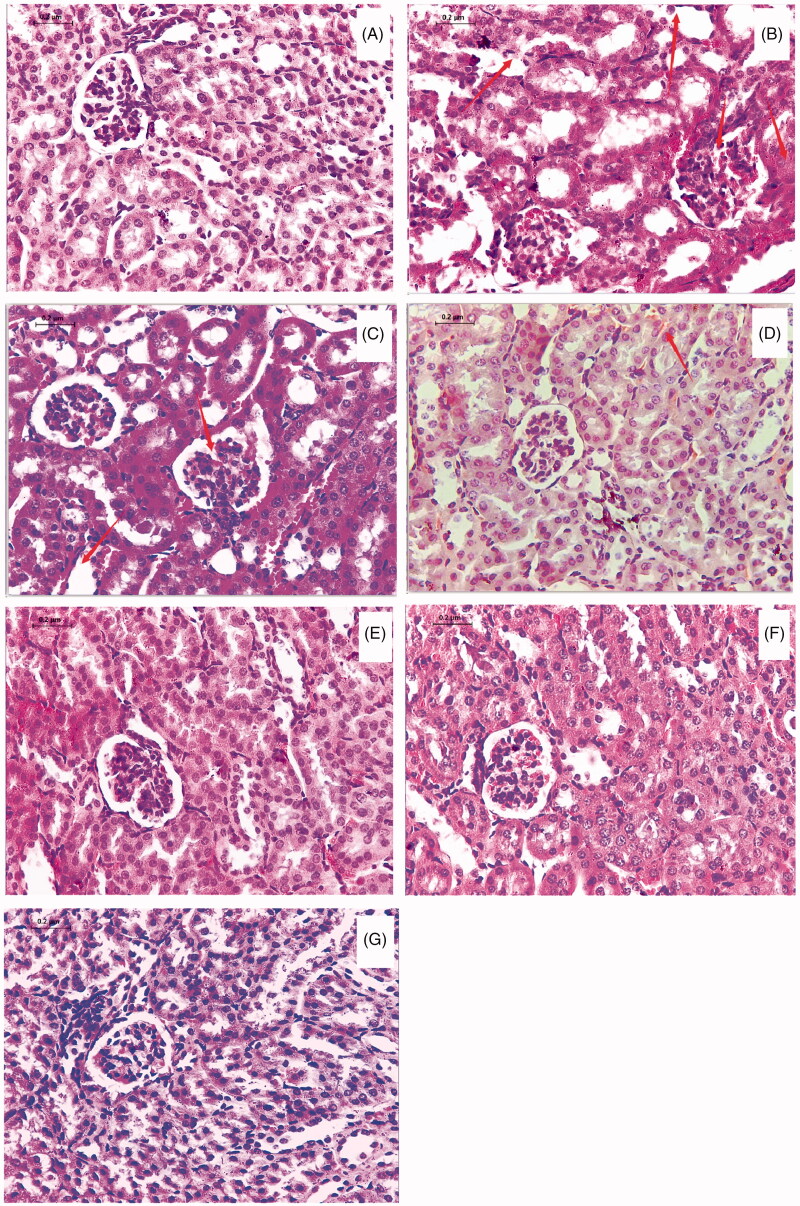
Morphological examination of the kidneys (HE, 400×). (A) NC control; (B) MC group; (C) AP group; (D) EGCG group; (E) EGCG + Vc group; (F) EGCG + glycerol group; (G) EGCG + Vc + glycerol group.

Compared with the NC group, congestion, vacuolation, degenerated kidney tubules, and cell necrosis were observed in kidney of the MC group. There was no obvious improvement in renal damage of the AP group compared with the MC group. In the EGCG group, the renal tubule lumen still revealed vacuolation, and the renal interstitial part revealed hyperaemia. Renal tubular epithelial cells in the EGCG + Vc and the EGCG + glycerol groups still revealed partial cell necrosis. Compared with the other groups, the tubular expansion of the EGCG + Vc + glycerol group restored and the morphology of the balloon and the renal tubule tended to be normal. This indicated that EGCG combined with Vc and glycerol could alleviate kidney damage of hyperuricemic mice (Figure 8).

## Discussion

EGCG is an important flavour and active component in green tea (Khan and Mukhtar 2007), but EGCG is not stable. Vc not only plays a good antioxidant role *in vivo*, but also has anticancer, immune regulation effects, etc. (Lee et al. 2002). It participates in various biochemical reactions *in vivo* as a component of coenzymes or prosthetic groups of various enzymes. It has been found that the addition of Vc to the catechins culture solution *in vitro* can improve the stability of catechins (Zhu et al. 2003). Glycerol is a commonly used pharmaceutical excipient which could increase the permeation number of drugs.

This study showed that EGCG was very unstable under conditions of high temperatures, alkaline solutions or artificial simulated intestinal fluids. However, EGCG when used in combination with Vc and glycerol could remarkably enhance its stability. The EGCG recovery rate of free EGCG group was 32.34 ± 1.86% after incubated for 4 h at 80 °C, while the EGCG recovery rate of EGCG combined with Vc and glycerol group was up to 98.90 ± 0.64%. The EGCG recovery rate of free EGCG group was 1.11 ± 0.07% after it was incubated for 6 h at pH 8, yet the EGCG recovery rate of EGCG combined with Vc and glycerol group was up to 91.82 ± 5.13%. The EGCG recovery rate of free EGCG group was 5.44 ± 1.16% and 8.03 ± 2.10% after they were incubated for 4 h in artificial simulated intestinal fluid and artificial simulated blank intestinal fluid, but the EGCG recovery rate of EGCG combined with Vc and glycerol group was up to 88.85 ± 2.63% and 90.19 ± 5.12%, respectively. The results showed that EGCG when used in combination with Vc and glycerol could notably increase its stability *in vitro*.

UA is a purine metabolite as well as a natural antioxidant in the human body, which can clear away 60% of the free radicals from the human blood (Kurra et al. 2009). If UA concentration is too high in blood, it will trigger oxidative stress and cause serious damage to the body. This study further explored the effect of EGCG combined with Vc and glycerol on serum UA values of hyperuricemic mice. Our studies have confirmed that EGCG might possess a potent hypouricemic effect on the hyperuricemic mice through suppressing XOD and ADA activities and inhibit renal GLUT9 and URAT1 expression levels (Zhu et al. 2018). In the study, EGCG combined with Vc and glycerol was administrated intragastrically to hyperuricemic mice. The results showed that UA-lowering activity of EGCG combined with Vc and glycerol was better than that of EGCG, EGCG + Vc and EGCG + glycerol groups. Serum BUN and Cr values are important indicators of evaluating renal function (Hoffmann et al. 2010; Chen et al. 2013). High values of serum BUN indicated urea clearance reduction, and abnormally elevated of the serum Cr is related to kidney damage. XOD and ADA are the key enzymes of UA production (Borges et al. 2002; Belle et al. 2011). Compared with the model group, the serum BUN, Cr values and the activities of ADA and XOD of mice in each EGCG-administered group were significantly reduced, showing good renal protection. The serum BUN, Cr values and the activities of ADA and XOD of EGCG + Vc + glycerol group were significantly lower than that of other groups. The renal organic ion transporters GLUT9 and URAT1 were the key proteins of renal tubules that are responsible for uric acid re-absorption. ABCG2, and OAT1/2 are three transporters related to uric acid secretion (Sato et al. 2010; Chen et al. 2015; Woodward 2015).

EGCG combined with Vc and glycerol remarkably down-regulated the gene expression levels of GLUT9 and URAT1, and up-regulated the gene expression levels of ABCG2, and OAT1/2. In addition, EGCG combined with Vc and glycerol could significantly improve kidney damage in hyperuricemic mice. The main reason might be that Vc has a good antioxidant effect *in vivo*, which may reduce the degradation or epimerization reaction of EGCG before intestinal absorption by reducing polyphenol free radicals. While glycerol may increase the viscosity of EGCG solution, enhance the sustained release of EGCG *in vivo*, and promote the absorption of EGCG in the small intestine.

The data presented here demonstrated that EGCG combined with Vc and glycerol could not only improve the stability of EGCG *in vitro*, but also had a synergistic effect on UA-lowering activity and renal function protection in hyperuricaemia mice. EGCG combined with Vc and glycerol is of great significance in the prevention and treatment of hyperuricaemia. In the future, we will continue to explore the effects of EGCG combined with Vc and glycerol on oral bioavailability of EGCG. How to further improve the stability and biological activities of EGCG requires further investigation.

## Conclusions

This study found that EGCG combined with Vc and glycerol could dramatically promote the stability of EGCG at high temperature and in alkaline environment, especially in the artificial simulated intestinal fluid. In addition, EGCG combined with Vc and glycerol could increase the effect of EGCG on lowering serum UA and improve the kidney damage obviously in hyperuricemic mice. Its mechanism might be through inhibiting the activity of ADA and XOD, down-regulating the mRNA expression levels of GLUT 9 and URAT 1 and up-regulated the gene expression levels of ABCG2, and OAT1/2. Therefore, the stability and biological activity of EGCG combined with Vc and glycerol were superior to free EGCG.
